# A reaction-diffusion model of the receptor-toxin-antibody interaction

**DOI:** 10.1186/1742-4682-8-32

**Published:** 2011-09-07

**Authors:** Vladas Skakauskas, Pranas Katauskis, Alex Skvortsov

**Affiliations:** 1 Faculty of Mathematics and Informatics, Vilnius University, 24 Naugarduko st., LT-03225, Vilnius, Lithuania; 2 HPP Division, Defence Science and Technology Organisation, 506 Lorimer st., VIC 3207, Melbourne, Australia

## Abstract

**Background:**

It was recently shown that the treatment effect of an antibody can be described by a consolidated parameter which includes the reaction rates of the receptor-toxin-antibody kinetics and the relative concentration of reacting species. As a result, any given value of this parameter determines an associated range of antibody kinetic properties and its relative concentration in order to achieve a desirable therapeutic effect. In the current study we generalize the existing kinetic model by explicitly taking into account the diffusion fluxes of the species.

**Results:**

A refined model of receptor-toxin-antibody (RTA) interaction is studied numerically. The protective properties of an antibody against a given toxin are evaluated for a spherical cell placed into a toxin-antibody solution. The selection of parameters for numerical simulation approximately corresponds to the practically relevant values reported in the literature with the significant ranges in variation to allow demonstration of different regimes of intracellular transport.

**Conclusions:**

The proposed refinement of the RTA model may become important for the consistent evaluation of protective potential of an antibody and for the estimation of the time period during which the application of this antibody becomes the most effective. It can be a useful tool for *in vitro *selection of potential protective antibodies for progression to *in vivo *evaluation.

## 1. Background

The successful bio-medical application of antibodies is well-documented (see [1,2] and references therein) and there is an ever-increasing interest in the application of antibodies for a mitigation of the effect of toxins associated with various biological threats (epidemic outbreaks or malicious releases) [3-5]. With the recent progress in bio-engineering, many antibodies with different affinity parameters have been generated. For a long time the main target of antibody design has been the antibody affinity. However, according to recent results [6], affinity, on its own, is a poor predictor of protective or therapeutic potential of an antibody. In fact, the treatment effect of an antibody can be described by a consolidated parameter which includes the reaction rates of the receptor-toxin-antibody kinetics and the relative concentration of reacting species [6]. As a result, any given value of this parameter determines an associated range of antibody kinetic properties and its relative concentration in order to achieve a desirable therapeutic effect. Analytical models, similar to those reported in [6], can be a useful tool for *in vitro *selection of potentially protective antibodies for progression to *in vivo *evaluation. They can significantly reduce the cost of research and development programs by optimizing associated experimental efforts. From this perspective, extension and validation of such models becomes an important goal for biomedical modelling which is partially addressed in the current study.

There are a number of ways of refining the simple kinetic model for the Receptor-Toxin-Antibody (RTA) system proposed in [6]. The possibilities include incorporating a mechanism of receptor recycling, complex pathways for toxin internalization or multiple receptor population [7]. The focus of our study is on incorporation of the diffusion effects in the theoretical framework of RTA, i.e. enhancement of the reaction RTA model [6] with the capability to account for the diffusion fluxes of reacting species [7]. Such enhancement not only enables the application of the RTA model in more realistic setting (i.e. instead of the simplified "well-mixed" approximation [6] the reaction-diffusive RTA model can describe propagation of toxin into a single cell or into a system of cells), but also provides a high fidelity estimation of the limiting uptake rate of toxin by a cell (especially when it is limited by diffusion). More importantly, the refined model allows consistent simulation of the so-called 'window of opportunity' (period of time after exposure to toxin when the application of an antibody is the most effective). We believe the two latter parameters (the limiting uptake rate and the 'window of opportunity') can become the key parameters in the optimization study for the future antibody design.

The incorporation of diffusion fluxes into the RTA model can be implemented based on a generalization of the well-known analytical framework for ligand-receptor binding [6-10]. From a mathematical point of view, the inclusion of diffusion terms into the RTA kinetic model leads to significant complications (system of nonlinear PDEs instead of system of ODEs), which usually prevent any analytical progress and implies numerical solutions. This was the main motivation for our approach to tackle the refined RTA model. The aim of this study is to numerically evaluate the protective properties of an antibody against a given toxin in the model of a spherical cell placed into a toxin-antibody solution. We consider the problem of the RTA interaction in the most general setting, when relative concentrations of species are arbitrary and all diffusive fluxes are taken into account (toxin, antibody and associated complexes). We calculate the antibody treatment efficiency parameter under various scenarios and identify the causes of time variation of this parameter.

We also study the RTA interaction in the 'Well-Mixed Solution' (WMS) model, i.e. when the solution of a toxin, antibody, and toxin-antibody complex is assumed to be uniformly mixed and homogeneously distributed in an extracellular space. In this case all diffusion fluxes disappear and the model can be described by Ordinary Differential Equations (ODE). It is worth noting that, since in such approach receptors are still confined to the single cell surface, our model is different from the "well-mixed" model proposed in [6] where all species are homogeneously distributed over the whole space. But in the case of a low internalization rate (i.e. low toxin inflow into a cell) the governing equations of these models are of the same type.

The paper is organized as follows. In Section 3 we introduce the reaction-diffusion model for RTA. The WMS model is presented in Section 4. The results are presented in Section 5. Conclusions and summarising remarks are presented in Section 6.

## 2. Notation

Ω - the extracellular domain, i.e. the problem domain where species diffuse and react (i.e. toxin, antibody, and toxin-antibody complex),

*S_e _*- the external surface of Ω,

*S_c _*- the cell surface (inner surface of Ω),

*r*_0 _- the concentration of receptors on the cell surface,

*θ*(*t*, **x**) - the the fraction of bounded receptors,

*r*_0_*θ *- the concentration of the toxin-bound receptors (confined to *S_c_*),

*r*_0_(1 - *θ*) - the concentration of free receptors,

*u_T_*, *u_A_*, and *u_C _*- the concentrations of toxin, antibody, and toxin-antibody complex,

$u_{T}^{0},u_{A}^{0},u_{C}^{0}$- the initial concentrations,

*κ_T_*, *κ_A_*, and *κ_C _*- the diffusivities of the toxin, antibody, and toxin-antibody complex,

*k*_1_, *k*_-1 _- the forward and reverse constants of toxin-antibody reaction rate,

*k*_2 _and *k*_-2 _- the forward and reverse constants of toxin and receptor binding rate,

*k*_3 _- the rate constant of toxin internalization,

*∂_n _*- the outward normal derivative on *S_e _*or *S_c_*,

*∂_t _*= *∂/∂t*,

Δ - the Laplace operator,

*ψ*(*t*) - the antibody protection factor (a relative reduction of toxin inside a cell due to application of antibody).

## 3. Reaction-Diffusion Model for RTA Interaction

The reaction-diffusion system for the RTA interaction can be derived based on well-known results of the receptor-ligand system (law of mass action and diffusion). By including antibody into the system we arrive at the following equations

(1) $$\left\{\begin{array}{c} \partial_{t}u_{T}=-k_{1}u_{T}u_{A}+k_{-1}u_{C}+\kappa_{T}\Delta u_{T},\;x\in \Omega ,\;t>0,\\ u_{T}{|}_{S_{e}}=u_{T}^{0},\;t>0,\\ \partial_{n}u_{T}=\frac{r_{0}}{\kappa_{T}}\left(-k_{2}\left(1-\theta \right)u_{T}+k_{-2}\theta \right),\;x\in S_{c},\;t>0,\\ u_{T}{|}_{t=0}=u_{T}^{0},\;x\in \Omega , \end{array}\right)$$

(2) $$\left\{\begin{array}{c} \partial_{t}\theta =k_{2}\left(1-\theta \right)u_{T}-k_{-2}\theta -k_{3}\theta ,\;x\in S_{c},\;t>0,\\ \theta {|}_{t=0}=0,\;x\in S_{c}, \end{array}\right)$$

(3) $$\left\{\begin{array}{c} \partial_{t}u_{A}=-k_{1}u_{T}u_{A}+k_{-1}u_{C}+\kappa_{A}\Delta u_{A},\;x\in \Omega ,\;t>0,\\ u_{A}{|}_{S_{e}}=u_{A}^{0},\;t>0,\\ \partial_{n}u_{A}{|}_{S_{c}}=0,\;t>0,\\ u_{A}{|}_{t=0}=u_{A}^{0},\;x\in \Omega , \end{array}\right)$$

(4) $$\left\{\begin{array}{c} \partial_{t}u_{C}=k_{1}u_{T}u_{A}-k_{-1}u_{C}+\kappa_{C}\Delta u_{C},\;x\in \Omega ,\;t>0,\\ u_{C}{|}_{S_{e}}=0,\;t>0,\\ \partial_{n}u_{C}{|}_{S_{c}}=0,\;t>0,\\ u_{C}{|}_{t=0}=0,\;x\in \Omega . \end{array}\right)$$

We disregard any excretion mechanism since we assume that it is nonsignificant over the time scales of interest (i.e. internalization time, time of toxin depletion etc).

The boundary conditions at the system above correspond to a case where initially the toxin and antibody are distributed homogeneously in the extracellular domain Ω. The boundary conditions on the outer boundary of the domain are assumed to be the constant concentrations of toxin and antibody and zero concentration of toxin-antibody complex. It is worth noting that in this case the gradients of *u_T_*, *u_A_*, *u_C _*are nonzero at the outer surface of the domain and they provide a time-dependent influx of species into Ω (with implication no conservation law for *u_T_*, *u_A_*, *u_C_*). Indeed, in such an approach we disregard any depletion of toxin and antibody within Ω (the depletion will be taken into account in the WNS model, see below). In a practical experiment this setup can correspond to a single cell embedded into a large volume (compartment) of toxin-antibody solution, so toxin and antibody are in excess. In this context it is also worth noting that in the real biomedical scenarios the concentration of toxin is usually very low with respect to the concentration of receptor due to the high concentration of receptors on the surface of living cells and the high toxicological effect (lethal dose) of the most toxins of interest. This implies that the condition of the excess of antibody over toxin is practically relevant and are very easy to achieve (e.g. see experimental results of [11], where the concentration of ricin was about a thousand times less than the concentration of antibody), while the condition of the excess of toxin over receptor seems to be infeasible for any *in vivo *situation (but the latter condition still can be used in lab experiments for the model validation).

It is worth mentioning that models similar to (1)-(4) have been extensively studied in application to biouptake of pollutants by micro-organisms, cellular nutrition, heterogeneous catalysis and analytical instrumental measurements (for comprehensive review of these studies see [12-17], and references therein). Equations (1)-(4) can be presented in non-dimensional form by using scales of τ_* _(time), *l *(length), and u__*__(concentration). By substituting new variables, $x=l\bar{x}$, $t=\tau_{*}\bar{t}$, $r_{0}=lu_{*}{\bar{r}}_{0}$, $u_{T}=u_{*}{ū}_{T}$ , $u_{A}=u_{*}{ū}_{A}$, $u_{C}=u_{*}{ū}_{C}$, $u_{T0}=u_{*}{ū}_{T}^{0}$, $u_{A0}=u_{*}{ū}_{A}^{0}$, ${\bar{k}}_{1}=\tau_{*}u_{*}k_{1}$, ${\bar{k}}_{2}=\tau_{*}u_{*}k_{2}$, ${\bar{k}}_{-1}=\tau_{*}k_{-1}$, ${\bar{k}}_{-2}=\tau_{*}k_{-2}$, ${\bar{k}}_{3}=\tau_{*}k_{3}$, ${\bar{\kappa }}_{T}=\tau_{*}\kappa_{T}l^{-2}$, ${\bar{\kappa }}_{A}=\tau_{*}\kappa_{A}l^{-2}$, ${\bar{\kappa }}_{C}=\tau_{*}\kappa_{C}l^{-2}$ into (1)-(4) we can deduce the same system, but only in non-dimensional variables. Therefore, for simplicity in what follows, we treat system (1)-(4) as non-dimensional.

The main parameter of interest is the antibody protection factor (a relative reduction of toxin attached to a cell due to application of antibody). This parameter can be defined by the following expression [6]

(5) $$\psi \left(t\right)=\frac{\int_{S_{c}}\theta {|}_{u_{A}^{0}>0}\text{d}S}{\int_{S_{c}}\theta {|}_{u_{A}^{0}=0}\text{d}S}.$$

By definition 0 ≤ *ψ *≤ 1 with the lower values of *ψ *corresponding to the more profound therapeutic effect of antibody treatment.

By employing (5) it is possible to derive a simple estimation for the saturation value of parameter *ψ *(i.e. for the limit *t *→ ∞). Indeed, from (1)-(4) for the steady-state limit we can write

(6) $$\theta =\theta^{\text{sat}}=\frac{k_{2}u_{T}^{\text{sat}}}{k_{2}u_{T}^{\text{sat}}+k_{-2}+k_{3}}=\frac{u_{T}^{\text{sat}}}{u_{T}^{\text{sat}}+K_{2}+b},$$

where $u_{T}^{\text{sat}}$ is the saturation concentration of toxin, *K*_2 _= *k*_-2_*/k*_2_, *b *= *k*_3_*/k*_2_. Then (5) leads to *ψ*_1 _= *ψ*^sat ^where

(7) $$\psi^{\text{sat}}=\frac{\theta^{\text{sat}}{|}_{u_{A}^{0}>0}}{\theta^{\text{sat}}{|}_{u_{A}^{0}=0}}.$$

So that *ψ*_1 _can be expressed in terms of only one 'bulk' variable $u_{T}^{\text{sat}}\geq 0$. Indeed, the value of *ψ*^sat ^can be appreciably affected by the diffusivities of species, since *κ_T_*, *κ_A_*, *κ_C _*determine the saturation value $u_{T}^{\text{sat}}$ by virtue of Eqs. (1)-(4).

## 4. WMS Model for RTA Interaction

The WMS model corresponds to an assumption that all species (toxin, antibody, and toxin-antibody complex) are distributed uniformly within the domain Ω. This implies no spatial gradients of concentrations, so all diffusivity terms disappear from system (1)-(4). Contrary to (1)-(4) we also assume that there are no fluxes of species across *S_e_*, so we account for depletion of species in the cell compartment Ω (a simple yet consistent approach that accounts for the depletion effect was proposed in [17]). The process of toxin internalization (i.e. flux of toxin through the cell surface) can be modelled in this case as a given rate of toxin removal from the whole system [9]. Then the WMS model can be translated to a system of ODEs:

(8) $$\left\{\begin{array}{c} {\overset{{}^{\circ}}{u}}_{T}=-k_{1}u_{T}u_{A}+k_{-1}u_{C}-k_{4}r_{0}\left(k_{2}\left(1-\theta \right)u_{T}-k_{-2}\theta \right),\;t>0,\\ u_{T}{|}_{t=0}=u_{T}^{0}, \end{array}\right)$$

(9) $$\left\{\begin{array}{c} \overset{{}^{\circ}}{\theta }=k_{2}\left(1-\theta \right)u_{T}-k_{-2}\theta -k_{3}\theta ,\;t>0,\\ \theta {|}_{t=0}=0, \end{array}\right)$$

(10) $$\left\{\begin{array}{c} {\overset{{}^{\circ}}{u}}_{A}=-k_{1}u_{T}u_{A}+k_{-1}u_{C},\;t>0,\\ u_{A}{|}_{t=0}=u_{A}^{0}, \end{array}\right)$$

(11) $$\left\{\begin{array}{c} {\overset{{}^{\circ}}{u}}_{C}=k_{1}u_{T}u_{A}-k_{-1}u_{C},\;t>0,\\ u_{C}{|}_{t=0}=0. \end{array}\right)$$

Here a dot is placed over the variables to represent a time derivative; *k*_4 _= *S_c_/V*_Ω_, where *S_c _*and *V*_Ω _are the area of cell and the extracellular volume. For instance, for a spherical cell of radius *ρ_c_*, *V*_Ω _is a domain between the cell and a concentric sphere of radius *ρ_e _*>*ρ_c_*, $V_{\Omega }=\frac{4}{3}\pi \left(\rho_{e}^{3}-\rho_{c}^{3}\right)$, $S_{c}=4\pi \rho_{c}^{2}$, and $k_{4}=3\rho_{c}^{2}/)\left(\rho_{e}^{3}-\rho_{c}^{3}\right)$. For a simple model of cell culture (a uniformly distributed system of cells) the average density of cell distribution, *n*, is approximately equal to $3∕\left(4\pi \rho_{e}^{3}\right)$, so we can treat the 'external' scale *ρ_e _*as the size of a compartment occupied by an individual cell in the culture. From this perspective, the dependence of *ψ*(*ρ_e_*) presented below can provide insight into the dependence of *ψ *on the cell packing density in the culture since *ρ_e _*≈ [3/(4π*n*)]^1/3 ^(see below).

The WMS model (8)-(11) is worth comparing with the model of the RTA interaction proposed in [6] (a kinetic model of uniformly distributed chemical species and cells). Despite these models being essentially different in their geometrical setting (in our case the receptors are still confined to a surface of a single cell), their governing equations become similar in the case when toxin inflow into a cell can be neglected (i.e. low internalization rate); the latter case seems to be very typical for many practical situations [7]. The WMS model (8)-(11) being a system of ODEs is much easier to analyze and solve numerically than the full RTA model (1)-(4) but indeed the WMS model cannot be used for estimating the effect of diffusivity of species on the protective properties of antibody (since it contains no diffusivity parameters).

With toxin internalization taken into account, the WMS model has only one conservation law $u_{C}+u_{A}=u_{A}^{0}$ (internalization implies that toxin is gradually taken away from the system). However, in the case of the low internalization rate we can set *k*_3 _= 0 and also deduce an "approximate" conservation law for toxin, viz., $u_{T}+u_{C}+k_{4}r_{0}\theta =u_{T}^{0}$, which is similar to one used in [6]. These conservation laws significantly simplify an analytical treatment of the WMS model. For instance, from Eqs. (7) and (8)-(11) it is possible to derive an approximate analytical expression for the saturation value of protection factor *ψ*^sat^. Actually, for the steady-state solution of system (8)-(11) without internalization rate (*k*_3 _= 0) it is straightforward to derive the following closed equation

(12) $$\left(1-\theta \right)\left(u_{T}^{0}-R_{0}\theta -\frac{\varepsilon u_{A}^{0}\theta }{1+\left(\varepsilon -1\right)\theta }\right)=K_{2}\theta ,$$

where ε = *K*_2_/*K*_1_, *K*_1 _= *k *_-1_/*k*_1_, *K*_2 _= *k *_-2_/*k*_2_, *R*_0 _= *r*_0_*k*_4 _(the same equation is given in [6] for the "well mixed" model). Then the solution of this equation enables the calculation of protection factor *ψ*_2 _= *ψ*^sat ^by means of Eq. (7).

We solve Eq. (12) numerically and compare the numerical results with the approximate analytical predictions deduced from the asymptotic solutions of Eq. (12). Some asymptotic analysis of Eq. (12) is presented in [6]. Our range of parameters corresponds to the case $R_{0}∕\left(\varepsilon u_{A}^{0}\right)\ll 1$ and this enables derivation of the approximate formula

(13) $$\psi^{\text{sat}}\approx \psi_{3}=\frac{F\left(u_{A}^{0},u_{T}^{0}\right)}{F\left(0,u_{T}^{0}\right)},$$

where $F\left(x,y\right)=\left(q_{1}-\sqrt{q_{1}^{2}-4q_{2}y}\right)∕\left(2q_{2}\right)$, *q*_1 _= *K*_2 _+ *εx *- (*ε *- 2)*y *and *q*_2 _= *q*_1 _- (*εK*_2 _+ *y*).

In order to verify our estimation of *ψ *near the saturation limit, we also solved non-steady system (8)-(11) numerically for large time and then by employing formula (7) determined function *ψ*_4 _= *ψ*^sat^. Table 1 shows that for the practically important cases the expressions for *ψ*_2_, *ψ*_3_, and *ψ*_4 _are in the very good agreement. Table 1 also demonstrates *ψ*^sat ^for the case where internalization rate is taken into account.

**Table 1:** Comparison of saturation values of *ψ *for WMS model: *ψ*^sat ^= *ψ*_2 _(12) and (7), *ψ*^sat ^= *ψ*_3 _(13), and *ψ*^sat ^= *ψ*_4_, where *ψ*_4 _is estimated from the solution of (8)-(11) and (7) at *t *= 10 000 s

*k*_1_	*k*_2_	*ψ*_2_	*ψ*_3_	*ψ*_4_	
				
				*k*_3 _= 0	*k*_3 _= 0.000033
0.013	0.0125	0.215524	0.215524	0.216026	0.206474
0.013	0.025	0.345686	0.345708	0.345903	0.332632
0.013	0.05	0.508760	0.508754	0.508767	0.493704
0.13	0.0125	0.027219	0.027220	0.027426	0.025913

## 5. Numerical Results

We treated system (1)-(4) numerically for the spherically symmetric domain *ρ *∈ [*ρ_c_, ρ_e_*] and *t *> 0 with an implicit finite-difference scheme [18]. These settings constitute the standard spherical cellular model [8-10,15]. Our selection of the values of parameters for the model (1)-(4) was motivated by the values available in the literature [11,19-22] with the extended range to allow exploration and illustration of the various transport regimes that are possible in the RTA system. If for some parameters (i.e. diffusivity) data were not available, then we used values from similar models [7-9] and added some ranges to cater for data uncertainty and to provide sensitivity analysis. The following values were used in most calculations [7]: u_* _= 6.02 · 10^13 ^cm^-3^, τ_* _= 1 s, *r*_0 _= 1.6 · 10^4^/*S_c_*, where 1.6 · 10^4 ^is the total number of receptors of the cell, *l *= 10^-2 ^cm, $S_{c}=4\pi \rho_{c}^{2}=4\pi \cdot 10^{-6}\text{ c}{\text{m}}^{2}$, ${\bar{r}}_{0}=2.115\cdot 10^{-3}$. The values of the other parameters are given in Table 2. If values of *k*_1_, *k*_2_, *κ_A_*, and *κ_T _*differ from those given in Table 2, they are specified in the legends of plots. We expect that the chosen values of parameters were representative enough to illustrate a rich variety of possible scenarios of the evolution of the RTA system and provide a reasonable estimate of timescales of the associated dynamics. The consistent match of the numerical predictions with the specific experimental results (i.e. on the ricin-neutralising antibodies [11,19-21]) would involve some additional assumptions about the relationship between the concentration of species and observable parameters (e.g. cellular viability) and was outside of the scope of the current paper.

**Table 2:** Values of parameters used in calculations

Parameter	Dimensional value	Non-dimensional value
*k*_1_	1.3 ·10^5 ^M^-1 ^s^-1^	1.3 · 10^-2^
*k*_2_	1.25 ·10^5 ^M^-1 ^s^-1^	1.25 · 10^-2^
*k*_-1_	1.4 ·10^-4 ^s^-1^	1.4 · 10^-4^
*k*_-2_	5.2 ·10^-4 ^s^-1^	5.2 · 10^-4^
*k*_3_	3.3 ·10^-5 ^s^-1^	3.3 · 10^-5^
*κ_T_*	10^-6 ^cm^2 ^s^-1^	10^-2^
*κ_A_*	10^-6 ^cm^2 ^s^-1^	10^-2^
*κ_C_*	10^-6 ^cm^2 ^s^-1^	10^-2^
*ρ_c_*	10^-3 ^cm	10^-1^
*ρ_e_*	2 · 10^-3^, 5 · 10^-3 ^cm	2, 5
$u_{A}^{0}$	6.02 · 10^-13 ^cm^-3^	1
$u_{T}^{0}$	3.01 · 10^-13^, 6.02 · 10 ^-14 ^cm^-3^	0.5, 0.1

**Figure 1:**
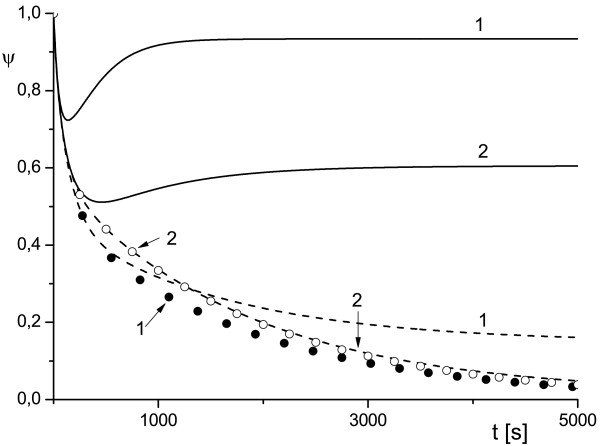
**Effect of variation of the scale of cell compartment and toxin diffusivity on protection factor**. External radius of the cell compartment *ρ_e _*= 2 (1) and *ρ_e _*= 5 (2), *κ_T _*= 10^-2 ^(solid line), *κ_T _*= 10^-3 ^(dashed line), *κ_T _*= 10^-4 ^(symbols) and $u_{T}^{0}=0.5$.

**Figure 2:**
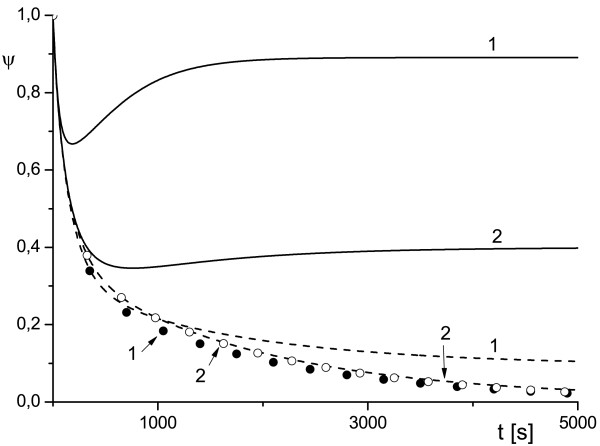
**Effect of variation of the scale of cell compartment and toxin diffusivity on protection factor**. External radius of the cell compartment *ρ_e _*= 2 (1) and *ρ_e _*= 5 (2), *κ_T _*= 10^-2 ^(solid line), *κ_T _*= 10^-3 ^(dashed line), *κ_T _*= 10^-4 ^(symbols) and $u_{T}^{0}=0.3$.

**Figure 3:**
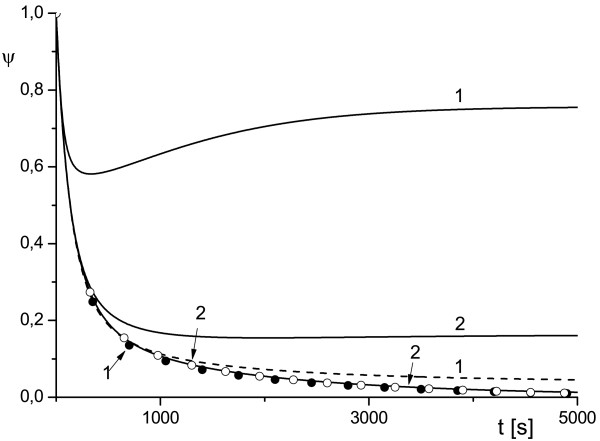
**Effect of variation of the scale of cell compartment and toxin diffusivity on protection factor**. External radius of the cell compartment *ρ_e _*= 2 (1) and *ρ_e _*= 5 (2), *κ_T _*= 10^-2 ^(solid line), *κ_T _*= 10^-3 ^(dashed line), *κ_T _*= 10^-4 ^(symbols) and $u_{T}^{0}=0.1$.

**Figure 4:**
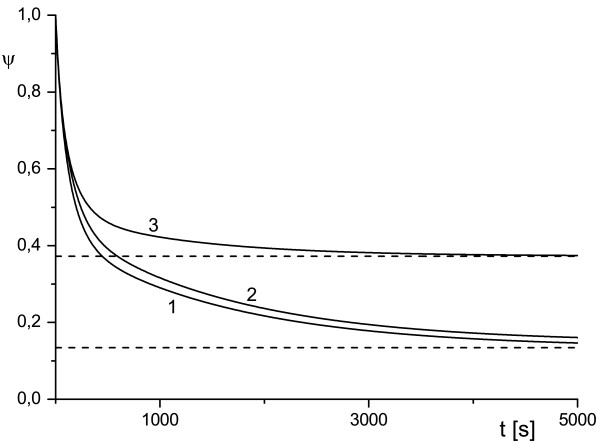
**Effect of the antibody diffusivity on the antibody protection factor**. Antibody diffusivity *κ_A _*= 10^-1 ^(1), *κ_A _*= 10^-2 ^(2), *κ_A _*= 10^-3 ^(3). Horizontal lines correspond to values of *ψ*^sat ^given by Eq. (7) for curves 1 and 2.

**Figure 5:**
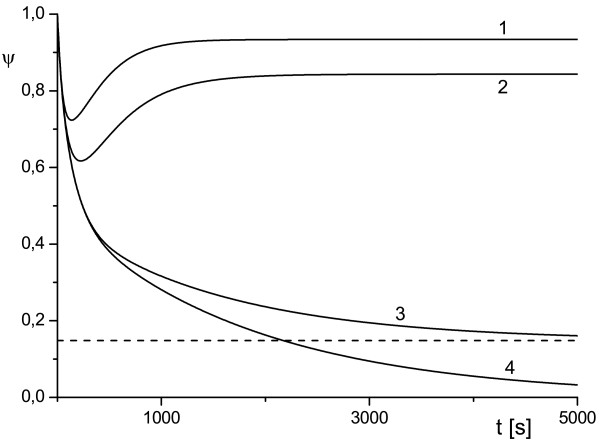
**Effect of toxin diffusivity on antibody protection factor**. Toxin diffusivity *κ_T _*= 10^-2 ^(1), *κ_T _*= 5 · 10^-3 ^(2), *κ_T _*= 10^-3 ^(3), *κ_T _*= 10^-4 ^(4). Horizontal line corresponds to value of *ψ*^sat ^given by Eq. (7) for curve 3.

**Figure 6:**
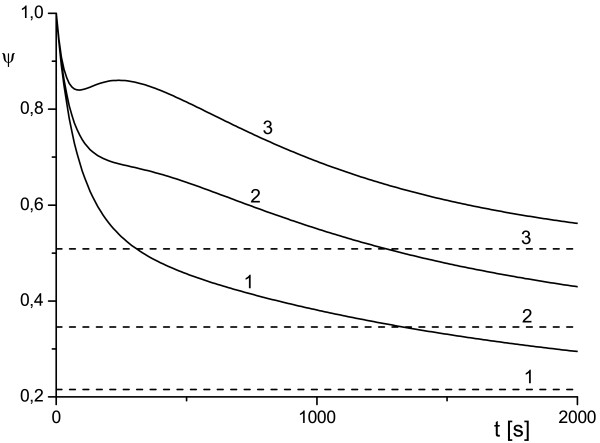
**Behavior of antibody protection function determined by WMS model for large time**. Plots demonstrate convergence of *ψ *to saturation limit for different values of parameters *k*_1 _and *k*_2 _at *ρ_e _*= 2; *k*_1 _= 1.3 · 10^-2^, *k*_2 _: 1.25 · 10^-2 ^(1), 2.5 · 10^-2 ^(2), 5 · 10^-2 ^(3). Horizontal lines correspond to values of *ψ*^sat ^given by (13).

**Figure 7:**
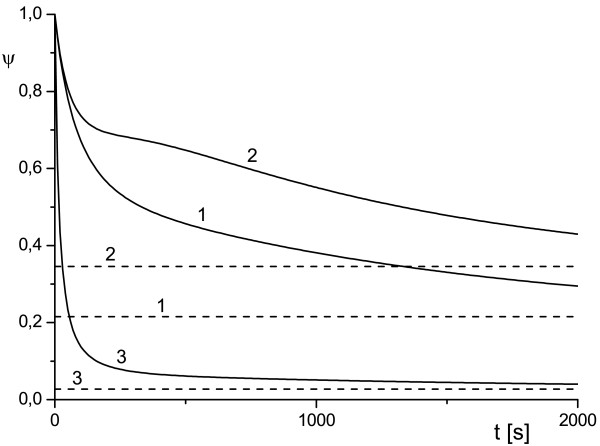
**Behavior of antibody protection function determined by WMS model for large time**. Plots demonstrate convergence of *ψ *to saturation limit for different values of parameters *k*_1 _and *k*_2 _at *ρ_e _*= 2; *k*_1 _= 1.3 · 10^-2^, *k*_2 _= 1.25 · 10^-2 ^(1), *k*_1 _= 1.3 · 10^-2^, *k*_2 _= 2.5 · 10^-2 ^(2), *k*_1 _= 1.3 · 10^-1^, *k*_2 _= 1.25 · 10^-2 ^(3). Horizontal lines correspond to values of *ψ*^sat ^given by (13).

The results of the numerical solutions are presented in Figures 1, 2, 3, 4, 5, 6, 7 and Tables 1, 3. As we indicated in the Background, the main purpose of our study was to estimate the effect of diffusive parameters of the species on the protective properties of an antibody. As such, most plots are presented below to illustrate this effect.

**Table 3:** Comparison of saturation values of *ψ *for model (1)-(4): *ψ*^sat ^= *ψ*_1 _and *ψ*^sat ^= *ψ*_5_, where *ψ*_1 _is determined by (6) and (7) with $u_{T}^{\text{sat}}$ estimated by (1)-(4) at *t *= 1000 s, while *ψ*_5 _is determined by (7) with *θ*^sat ^estimated by (1)-(4) at *t *= 10 000 s

*κ_T _*(*κ*_*A *_= 10^-2^)	*ψ*_1_	*ψ*_5_	*κ_A _*(*κ*_*T *_= 10^-3^)	*ψ*_1_	*ψ*_5_
10^-2^	0.9339	0.9339	10^-1^	0.1342	0.1345
5 · 10^-3^	0.8433	0.8433	10^-2^	0.1480	0.1483
10^-3^	0.1480	0.1483	10^-3^	0.3729	0.3726
10^-4^	0.0047	0.0034	10^-4^	0.9801	0.9801

To provide insight into the relation between the diffusion transport and the protective properties of an antibody in the spherical cellular model, it is convenient to employ the theoretical framework that is well-established in ecology and electrochemistry (toxin uptake by microorganisms and performance of microelectrodes) (e.g., see [15-17] and references therein). According to [15], the steady-state flux of toxin towards a spherical cell can be estimated from the following expression

(14) $$J\left(t\right)=\Lambda^{-1}u_{T}\left(t\right),\;\Lambda =\left(\frac{1}{k_{3}K_{*}}+\frac{\rho_{c}}{\kappa_{*}}\right),$$

where Λ is the conductance of the system (flux-concentration ratio), *u_T_*(*t*) is the concentration of toxin on the outer boundary of Ω, viz. $u_{T}\left(t\right)\;=u_{T}^{0}$ for the boundary condition of constant concentration or $u_{T}\left(t\right)=u_{T}^{0}\text{exp}\left(-t∕\tau_{d}\right)$ for the no-flux boundary condition, κ_* _is the effective diffusion of the toxin, *τ_d _*is the depletion time of toxin in the bulk, K_* _= *R*_0_/(*R*_0 _+ *K*_1_) [6]. It can be seen that the parameter κ_* _and depletion time *τ_d _*(if the depletion of toxin is significant) become two 'aggregated' parameters that can be used to comprehensively characterize the influence of an antibody on toxin transport in the model of spherical cell.

The term κ_* _/*ρ_c _*in Eq. (14) represents the diffusive conductance and the term K_*_k_3 _represents the internalization conductance [15]. The ratio of the two terms is

(15) $$L=K_{*}k_{3}\rho_{c}∕\kappa_{*},$$

which is called bioavalability number [15] and can be used to characterized the regime of toxin uptake by the cell [15,16]. If *L *≪ 1 the uptake flux is fully controlled by the internalization process, while in the opposite case *L *≫1 it is controlled by diffusion. Note that for the case of ricin competitive binding to cell receptors and the mono-clonal antibody 2B11 the value of parameter *L *≈ 10^-2^, i.e. flux is mostly controlled by internalization process. Importantly, even in the case of diffusion dominated flux the transport of toxin can be characterized by a rich variety of regimes that are parameterized based on the so-called degree of lability, so these regimes correspond to the different asymptotical values of parameters κ_*_, *τ_d _*[15-17].

A detailed analysis of various regimes of diffusion controlled transport emerging in the spherical cellular model is outside the scope of the current paper, so we briefly present here only some key points that are relevant to the understanding of our numerical simulations (for details we refer the reader to [15-17]). It can be shown that the ratio *p *= κ_* _/*κ_T _*is always within the range 1 ≤ *p *≤ ∞ with the minimal value *p *= 1 corresponding to the diffusion transport of toxin without presence of antibody (i.e. κ_* _= *κ_T _*). The latter condition together with Eqs. (14) leads to a simple estimate for the long-time asymptote of the protection factor of antibody (5)

(16) $$\psi \left(t\right)\approx \psi_{*}\text{exp}\left(\gamma t\right),\;\psi_{*}=\frac{1+L_{0}}{1+L_{0}∕p},$$

where $\gamma =1∕\tau_{d}-1∕\tau_{d}^{0},\tau_{d}^{0}$ is the depletion time of toxin without antibody, *L*_0 _= K_* _*k*_3_*ρ_c_/κ_T_*.

Equation (16) is an analogue of expression (13) that accounts for diffusion effects and toxin depletion. We observe that, depending on a value of the parameter *γ*, the asymptotical behavior of the protection factor can be either zero (*γ *< 0), infinity (*γ *> 0) or non-zero constant (*γ *= 0). For the diffusion controlled flux, *L*_0 _≫ 1 and ψ_* _= *p *while for the kinetically controlled regime *L*_0 _≪ 1 and ψ_* _= 1. This implies that by changing the diffusivity of the reacting species (i.e. by introducing an antibody) it is possible (at least in theory) to control the behavior of the protection factor *ψ*(*t*). The case of the constant toxin influx (i.e. no depletion) simply corresponds to *γ *= 0. We observed most of these scenarios in our numerical simulations (see below).

An interesting (and not intuitively obvious) result of expression (16) is the general inequity ψ_* _≥ 1 (more precisely 1 ≤ ψ_* _≤ 1 + *L*_0_). This means that for the diffusion-controlled scenario and for the case when toxin depletion is not significant (e.g. for *γT *≪ 1), the introduction of an antibody can only *increase *the flux of toxin towards the cell. This result is a clear manifestation of a possible contribution of the antibody-toxin complex to the total toxin flux, described in [15-17]. Importantly, that for a case of the fast kinetics (situation when the reaction of antibody-toxin complexation is much faster than diffusion time of reacting species) the effective diffusivity κ_* _is reduced to the mean diffusivity [16,17]

(17) $$\kappa_{*}=\frac{u_{T}\kappa_{T}+u_{C}\kappa_{C}}{u_{T}+u_{C}},$$

that can be significantly different from the diffusivity of a toxin *u_T_*. It is worth noting that antibody diffusivity does not appear in this expression. The "limit of mean diffusivity" for κ_* _given by (17) occurs only for the system with *κ_T _*≠ *κ_C _*[16,17] (which is usually the case because of a difference in molecular weights).

Some analytical models for the calculation of the toxin depletion times $\tau_{d}^{0}$, *τ_d _*have been proposed [16]. They are quite involved, and for details, we refer the reader to the original publications. The results [16] clearly demonstrate that the parameter $\gamma =1∕\tau_{d}-1∕\tau_{d}^{0}$ in (16) can depend on the 'external' scale *ρ_e _*(i.e. size of the cell 'compartment') in a quite convoluted way. As was mentioned above, the scale *ρ_e _*can be approximately related to the packing density of cells in a culture (*ρ_e _*≈ [3/(4*πn*)]^1/3^), so plots *ψ*(*ρ_e_*) can be also interpreted as a simple qualitative illustration of the effect of variation in packing density *n*.

The plots *ψ*(*ρ_e_*) in Figures 1, 2, 3 depict the dependence of the antibody protection factor *ψ *on the radius of external surface *ρ_e _*(i.e. a size of the cell compartment) and on toxin diffusivity *κ_T_*. We calculated *ψ *for two values of *ρ_e _*(*ρ_e _*= 2 and *ρ_e _*= 5), for two values of *κ_T _*(*κ_T _*= 10^-2 ^and *κ_T _*= 10^-3^), and three values $u_{T}^{0}$ ($u_{T}^{0}=0.5$ Figure 1, $u_{T}^{0}=0.3$ Figure 2, $u_{T}^{0}=0.1$ Figure 3).

We believe that the analytical results (16) discussed above and the numerical examples similar to those presented in Figures 1, 2, 3 may be important for either the planning of experiments (especially in cell culture) or for the correct interpretation of experimental data, since they provide a simple estimation for the amplitude of the observable effect (protection factor) and for the timescale during which this effect can occur (~ 1/*γ*).

The results depicted in Figures 1, 2, 3 also provide an illustrative example of the main finding of our study: the time evolution of the protection factor *ψ*(*t*) may switch from monotonic to markedly non-monotonic behavior with a variation of diffusion parameters of the RTA model. This phenomenon is in line with the theoretical framework proposed in [15-17]) and was observed frequently in our simulations.

Following the well-established application of the concept of lability to the spherical cell model [15-17], an incorporation of diffusion effects into our model enabled the simulation of a new phenomenology, which may occur in the RTA system. For instance, with system (1)-(4) we were able to model competitive behavior of the reaction and diffusion fluxes. As was mentioned above, the latter often manifests itself in rather convoluted (non-monotonic) dependence of concentration of species and their diffusion coefficients, see Figures 4 and 5. The plots in Figures 4 and 5 depict a variety of scenarios for time evolution of *ψ *for the different diffusivity of toxin and antibody (other parameters were the same). We can clearly see a switch from monotonic to non-monotonic behavior as we decrease diffusivity of toxin *κ_T _*(Figure 5). The cases of non-monotonic behavior with a profound minimum of *ψ*(*t*) provide revealing examples of the practically important concept of a 'window of opportunity' discussed in the Background. Once the function *ψ*(*t*) moves far away from its minimal value, the 'blocking' effect of an antibody markedly decreases. We observe that the 'window of opportunity' is very scenario-dependent and it can be easily estimated from the plots similar to those presented in Figure 5.

The plots presented in Figures 1, 2, 3, 4, 5 illustrate detailed insights into the transport process associated with the different behavior of the protective function *ψ*(*t*) owing to the introduction of antibody into the RTA system. From these results we can see that the non-monotonic behavior is caused by the non-monotonic supply (transport) of toxin across the compartment to the cell surface; this transport can be affected by changing the diffusivity of species. The main conclusion from our numerical results is that the relative diffusivity of species can be used to control the effect of antibody treatment during a short time after the exposure to a toxin (usually a few minutes).

The plots presented in Figures 6 and 7 show the dependence of *ψ *on *k*_1 _and *k*_2 _for the WMS model and demonstrate a possible switch of *ψ *from monotonic to non-monotonic behavior as *k*_2 _grows. Calculations show that *ψ*(*t*) depends very weakly on *ρ_e _*and this an indication that the reactions in the surface layers around the cell (diffusive and reaction) provide the dominant contribution to the transport properties of the system. This result is depicted in Figures 6 and 7 where all curves are given for *ρ_e _*= 2 and *ρ_e _*= 5. As time increases, function *ψ*(*t*) tends to the diffusivity-dependent asymptote *ψ*^sat ^= *ψ*_1 _for system (1)-(4) and to the reaction-dependent asymptote *ψ*^sat ^= *ψ*_3 _for the WMS model irrespective of its short-time behavior (which is indeed controlled by (16)). The diffusive dependency of the saturation limit of the system (1)-(4) becomes evident if we recall that at the steady state, the flux of internalized toxin (i.e. flux across *S_c_*) should be compensated by the diffusion influx across the outer surface *S_e_*. In order to validate this asymptotic behavior we computed a rich set of scenarios. These results are presented in Figures 4, 5, 6, 7 and in Tables 1 and 3. In Table 3 we compare *ψ*_1 _and *ψ*_5 _where *ψ*_1 _is determined by (6) and (7) with $u_{T}^{\text{sat}}$ estimated by (1)-(4) at *t *= 1000 s, while *ψ*_5 _is determined by (7) with *θ*^sat ^estimated (1)-(4) at *t *= 10 000 s. We observe that function *ψ*(*t*) converges to an asymptotic value, but this convergence can be rather slow.

As was suggested by one of the anonymous referees, the observable strongly non-monotonic behavior of parameter *ψ*(*t*) in some of our modeling scenarios can possibly be explained by applying the concept of dynamic speciation to the formation of a toxin-antibody complex [15-17]). In the diffusion-controlled regime the dynamic speciation (i.e. the fast toxin-antibody kinetics over diffusion time) can lead to the significant contribution to the toxin flux towards the cell and (under condition *κ_C _*<*κ_T_*) can even cause a 'retardation' effect [15]. After some estimations we found this hypothesis quite reasonable. For a cell size of *ρ_c _≈ *10^-5 ^m the diffusion time is *τ_κ _≈ *0.2 s for *κ ≈ *1 *· *10^-9^ m^2^s^-1^. The estimation for equilibration time *τ_e _*was derived from the rigorous theoretical framework proposed in [23] for competitive binding system (application of this framework to the toxin-receptor and toxin-antibody binding can be found in [6]). Indeed the equilibration time *τ_e _*is a strong function of the toxin concentration; it rapidly decreases as the toxin concentration increases (reacting species can faster find each other to form a complex). If as a reference point we assume that the value of parameters correspond to the scenario of binding of ricin to receptor and to the antibody then for the toxin concentration *T *= 10 pM the reaction time is of order of 10 s. By further increasing the toxin concentration (five times in our simulations) it appears that we approach the transition threshold from the 'inert' to the 'dynamic' complex, so the toxin-antibody complex starts contributing to the diffusion flux. A more challenging task was to identify the scenarios where this additional contribution can be appreciable, since the total flux is mainly internalization-controlled. Nevertheless, if we recall that diffusivities and concentration of species are varied by an order of hundreds, then reaching the diffusion-controlled regime in some of our simulations looks quite feasible. A more detailed interpretation of our numerical results with the concept of dynamic speciation would require additional numerical calculations (careful estimations of equilibration time of complex for each scenario) and is outside of the scope of the current study.

## 6. Concluding Remarks

In summary, we have refined the RTA model developed in [6] by incorporating diffusion of reacting species in the extracellular space. By solving numerically the system of nonlinear PDEs of the model we managed to simulate a rich variety of reaction-diffusion processes that may occur in the RTA system. For various combinations of parameters (rates of reactions, diffusivity and initial concentrations) we estimated the effect of antibody on the toxin penetration into a cell and expressed the effect of the antibody treatment in terms of a non-dimensional protection factor (relative reduction of toxin concentration within a cell). We demonstrated that this factor can be a significantly non-monotonic function of time and its behavior depends on an interplay between diffusive and reaction processes in the RTA model. We also examined the time evolution of the protection factor and found that it eventually tends to a diffusivity-dependent asymptotic limit, but the convergence to this asymptote may take significant time. From this perspective, the refinement of the RTA model proposed in the present study becomes important for the consistent evaluation of protective potential of an antibody and for the estimation of the time period during which the application of this antibody becomes the most effective.

The selection of the rate constants for numerical simulations was motivated by data reported in the literature [11,19-22], with the significant ranges of variability to provide a simple sensitivity analysis for the system under consideration. The values for other constants (i.e. diffusivity) were selected based on similarity with other models [8-10]. The chosen values of parameters enable an illustrative demonstration of a rich variety of regimes of the evolution that could occur in the RTA system. These regimes are similar to ones occurring in electrochemistry and ecological studies (performance of microelectrodes and toxin uptake by microorganisms). Further validation of the proposed model with a particular set of experimental data on toxin-neutralising antibodies (e.g. [11,21]) would require a separate study. Such a study would include an application of a data fitting algorithm that accounts for the experimental data uncertainty as well as some additional assumptions about relationships of the model predictions (concentration of species, protection factor) with the observable quantities (i.e. cellular viability). The latter assumptions may significantly affect the experimental data fit and the evaluation of predictive skills of the proposed model. We will report on such study in a separate publication.

## 7. Competing interests

The authors declare that they have no competing interests.

## 8. Authors contributions

AS suggested a simplified model of the RTA system. VS extended the model and proposed a consistent analytical framework for the modeling and simulation of the RTA interaction. PK implemented the implicit finite-difference scheme for the numerical solution of the refined PDE system. All authors contributed to the writing of the article, and read and approved the final manuscript.
